# Disease in Adults and in Children

**Published:** 1889-03

**Authors:** 


### SELECTIONS.

### Disease in Adults and in Children.

Dr. W. H. Dickinson, in his introductory lecture on the differences between children and adults in regard to morbid action and the effects of treatment (*Lancet* Nov. 3, 1888), says that such of their diseases as are of the nature of growths, partake of the rate of progress which belongs to the period of development. This applies to the fibroses as well as to isolated tumors, though the fibroses are far more infrequent than afterward. Many inflammatory disorders, especially of the brain, are more common in childhood than later; but with regard to many inflammations the power of recovery is greater. Inflammations of the lungs are far more frequent and cause more deaths, but in the individual the prospect is better. Acute rheumatism is comparatively infrequent, but when it occurs it brings greater danger to the heart. Some febrile complaints are more frequent, and some less so, than afterward; but as a rule their proportionate mortality is less. There are special differences in the action of drugs, the most important of which is the greater influence of alcohol for good and evil. Finally, children respond to treatment —as they often succumb to disease—more readily than do older patients; so that with them our responsibility is greater.

